# Host-Microbial Interactions in Systemic Lupus Erythematosus and Periodontitis

**DOI:** 10.3389/fimmu.2019.02602

**Published:** 2019-11-12

**Authors:** Larissa Pessoa, Gajender Aleti, Saibyasachi Choudhury, Daniel Nguyen, Tina Yaskell, Yun Zhang, Weizhong Li, Karen E. Nelson, Leopoldo Luiz Santos Neto, Adriana C. P. Sant'Ana, Marcelo Freire

**Affiliations:** ^1^Department of Prosthodontics and Periodontics, Bauru School of Dentistry, University of São Paulo, Bauru, Brazil; ^2^Department of Genomic Medicine, J. Craig Venter Institute, La Jolla, CA, United States; ^3^Applied Oral Sciences, The Forsyth Institute, Cambridge, MA, United States; ^4^Department of Genomic Medicine, J. Craig Venter Institute, Rockville, MD, United States; ^5^Rheumatology Department, Medical Faculty, University of Brasília, Brasilia, Brazil

**Keywords:** cytokines, gingival crevicular fluid, oral-systemic, host-microbes, autoimmunity, periodontal diseases

## Abstract

**Background:** Systemic lupus erythematosus (SLE) is a potentially fatal complex autoimmune disease, that is characterized by widespread inflammation manifesting tissue damage and comorbidities across the human body including heart, blood vessels, joints, skin, liver, kidneys, and periodontal tissues. The etiology of SLE is partially attributed to a deregulated inflammatory response to microbial dysbiosis and environmental changes. In the mouth, periodontal environment provides an optimal niche for local and systemic inflammation. Our aim was to evaluate the reciprocal impact of periodontal subgingival microbiome on SLE systemic inflammation.

**Methods:** Ninety-one female subjects were recruited, including healthy (*n* = 31), SLE-inactive (*n* = 29), and SLE-active (*n* = 31). Patients were screened for probing depth, bleeding on probing, clinical attachment level, and classified according to CDC/AAP criteria with or without periodontal dysbiosis. Serum inflammatory cytokines were measured by human cytokine panel and a targeted pathogenic subgingival biofilm panel was examined by DNA-DNA checkerboard from subgingival plaque samples.

**Results:** The results showed significant upregulation of serum proinflammatory cytokines in individuals with SLE when compared to controls. Stratification of subject's into SLE-inactive (I) and SLE-active (A) phenotypes or periodontitis and non-periodontitis groups provided new insights into SLE pathophysiology. Ten proinflammatory cytokines were upregulated in serum of SLE-I only and one in SLE-A only. Four molecules overlapped in SLE-A and SLE-I. Anti-inflammatory cytokines included IL-4 IL-10, which were upregulated in SLE-I sera (but not SLE-A), controlling clinical phenotypes. Out of 24 significant differential oral microbial abundances found in SLE, 14 unique subgingival bacteria profiles were found to be elevated in SLE. The most severe oral pathogens (*Treponema denticola* and *Tannerella forsythia*) showed increase abundances on SLE-A periodontal sites when compared to SLE-I and healthy controls. Inflammation as measured by cytokine-microbial correlations showed that periodontal pathogens dominating the environment increased proinflammatory cytokines systemically.

**Conclusions:** Altogether, low-grade systemic inflammation that influenced SLE disease activity and severity was correlated to dysbiotic changes of the oral microbiota present in periodontal diseases.

## Introduction

The human microbiome is in constant interaction with the host, modulating health and disease phenotypes. We now appreciate that oral bacteria plays indispensable roles in shaping the systemic host physiological landscape and in dysbiosis. As part of the upper digestive tract, the oral cavity presents specific niches, such as gingival sulcus and gingival crevicular fluid (GCF), which in turn harbor commensal and pathogenic bacteria with potential impact to oral and systemic health. Through local activation of inflammation, oral pathogens have shown to worsen the burden of chronic diseases through time including, type 2 diabetes, premature labor, rheumatoid arthritis, systemic lupus erythematosus (SLE), Alzheimer's, cardiovascular conditions, and cancer (1–4).

Locally, the oral commensal flora and tissue inflammation evolved to develop a relationship of homeostasis. In dysbiosis, pathogens become dominant, including species previously clustered according to their pathogenesis into the “red complex”- *Tannerella forsythia, Porphyromonas gingivalis*, and *Treponema denticola* (5). In periodontitis, for example, continuous and unresolved inflammatory response affects tooth-supporting tissue structures (periodontal ligament, connective tissue, and bone) (1, 6). As one of the most common infectious diseases globally, the etiology of periodontitis is multifactorial, and the microbial-inflammation imbalance leads to bone resorption and consequent tissue tooth loss (7). Inflammation precedes tissue loss and the systemic impact of periodontal dysbiosis goes beyond the oral compartment.

Systemic lupus erythematosus (SLE) is a multisystem autoimmune disease with increasing incidence worldwide (8). The Lupus foundation of America estimates that 1.5 million Americans have SLE, and at least five million people worldwide have a form of lupus (9, 10). SLE affects mostly women and most people with the condition manifests the symptoms between ages of 15–44. The current incidence of SLE is 16,000 new cases per year and patients experience significant symptoms, such as pain, and fatigue, hair loss, cognitive issues, physical impairments, oral and vaginal mucosa manifestations, affecting every facet of their lives (11). According to the American College of Rheumatology (ACR), an array of clinical exams and positive serology are used to diagnose disease, including malar rash, discoid rash, arthritis, kidney disorder, anemia or leukopenia, abnormal antinuclear antibodies (ANA) and anti-DNA or antiphospholipid antibodies (12). In addition to molecular diagnosis, systemic lupus erythematosus disease activity index (SLEDAI) is used in the assessment of disease severity and response to treatment; manifestations of comorbidities and molecular abnormalities affecting multiple organs of the body often challenges diagnostics at early stages (13). Although complex diseases have shown to be influenced by genetic and environmental forces, the gut microbiome, and recently the oral microbiome, showed direct impact on SLE subjects (14), the complexity of chronic diseases such as SLE is beyond isolated body compartments, and it requires the integration of host-microbial interactions. The heterogeneity of disease presentation and organ involvement contribute to clinical challenges for diagnosis and effective management (15). While several studies reported associations among human oral microbiota compositions in SLE (16–19), co-occurrences of specific periodontal pathogens and inflammatory cytokines important for low grade inflammation and chronic disease remains to be explored (2).

Studies have suggested that specific cytokines (elastase, interleukin -IL-1, IL-2, IL-6, IL-13, IFN-γ, TNF-α) were increased while others are decreased (IL-1β, IL-18) in either GCF or serum of SLE subjects (20–22), and similar alterations were observed in periodontitis patients (23). These subjected also showed elevated levels of proinflammatory cytokines (especially, IL-1 β and IL-6). In fact cytokines, such as internferon-IFN-γ, IL-10, IL-17, and IL-4, were increased in periodontitis compared to SLE, independent of their periodontal condition (23). The overexpression of IFN-α inducible genes were related to SLE flare rather than infection and the cytokine levels could be used to differentiate disease flare from infectious conditions (24). No study has previously investigated the combinatorial role of pro and anti-inflammatory cytokines in SLE patients compared to systemically healthy patients, both with periodontitis.

In this study we have investigated SLE phenotypic differences, oral microbiota associations with systemic inflammation to unravel forces shaping oral and broader systemic health. As such, we compared the subgingival microbial signatures from SLE and healthy controls to investigate host-microbial relationships. Specifically, we first sought to establish if specific microbial compositions correlated to periodontal clinical phenotypes with systemic levels of inflammatory cytokines. The presence of periodontal disease was assessed using categories of local inflammation, clinical attachment levels and bone loss, while SLEDAI > or <2 was assessed to categorize the individuals into SLE-active, SEL-inactive. The results indicate that chronic periodontitis and SLE present low-grade inflammation modulated periodontal diseases and specific microbial signatures. The association of subgingival microbial profiles with SLE and its association with periodontal clinical status and inflammatory markers established novel links developing a new framework for oral-systemic studies.

## Materials and Methods

### Subjects

Subjects were recruited at the Center of Rheumatology at the University Hospital in Brasília from July 2013 to March 2014, and samples were obtained with informed consent of the subjects. The diagnosis of SLE was initially made by the primary care physician and confirmed by rheumatologist specialist following guidelines of the American Academy of Rheumatology (according to ACR 1982/1997 revised classification criteria) (25, 26). This research project was approved by the Bauru Dental School-USP Ethics Committee of Research (protocol # 111.718) and by the Ethics Committee of Research. Subjects with antimicrobial treatments within 3 months of the start of the study were excluded. Clinical parameters for SLE and periodontal status were measured as follows:

#### SLE Status

SLE activity and diagnosis was investigated according to SLEDAI classification (27). SLE activity was determined by SLEDAI >2. Disease severity is measured according to SLICC/ACR-DI (Systemic Lupus International Collaborating Clinics of American College of Rheumatology Damage Index) proposed by Gladman et al. (28), which was used as an additional method of information on disease severity to better characterize the studied population. SLE patients were subdivided into two groups based on their disease activity: active (SLE-A; SLEDAI >2; *n* = 29), and inactive (SLE-I; SLEDAI ≤2; *n* = 31). The control group was composed of 31 systemically healthy women recruited at the University Hospital in Brasília during the same time period. All patients answered a health questionnaire investigating medical and dental history. A visual investigation of the oral cavity was performed at enrollment to investigate the number of remaining teeth, other oral diseases and periodontal health.

#### Periodontal Status

After recruitment of subjects, participants were referred to the Dental School of Brasília (Brasilia, Brazil) for clinical periodontal examination and sample collection. The clinical periodontal examination was performed by a single trained examiner before the collection of subgingival plaque and blood samples prior to oral and non-surgical periodontal treatment. The following parameters were investigated by using a UNC-15 millimeter periodontal probe at 6 sites/tooth: pocket probing depth (PD), clinical attachment level (CAL), and gingival bleeding index (29). Plaque index was assayed by staining the dental plaque with disclosing solution at mesial, buccal, distal and lingual sites (30). Periodontitis was defined as the presence of interproximal loss of attachment ≥3 mm and PD ≥4 mm in at least 2 sites of different tooth, according to CDC/AAP criteria (31, 32). After radiographic and clinical examination followed by collection of blood, subgingival plaque samples, patients were submitted to non-surgical periodontal treatment consisted of supra and subgingival scaling and root planing, when necessary, dental polishing, and oral hygiene instruction.

### Sample Collection

Subgingival plaque was collected prior to any treatment for all subjects, including non-surgical periodontal treatment. Subgingival plaque samples were collected from four sites showing the deepest probing depth in each patient. The area was isolated with cotton rolls to prevent contact with saliva and air dried. Supragingival plaque was removed with Gracey curettes previous to subgingival plaque collection. The subgingival plaque was retrieved by the introduction of PerioPaper strips into gingival sulcus for 30 s and stored in sterilized tubes at −80°C till processing. Blood samples were collected after routine examination for SLE monitoring. Control subjects were asked to perform similar examinations at the Hospital. PerioPaper strips were centrifuged at 3,000 × g for 15 min at 4°C in PBS elution for the dosage of the tissue destruction and bone resorption markers by Multiplex (Human MAP, Millipore, USA). The eluted samples were stored at −80°C up to processing.

### DNA Extraction, Amplification, and Checkerboard Measurements

DNA-DNA checkerboard hybridization was performed using the method developed by Socransky et al. (5). Briefly, to prepare standard probes, 40 selected bacterial species were grown on agar plates and were suspended in 1 mL of TE buffer (10 mM Tris-HCl, 0.1 mM EDTA, pH 7.6). Cells from subgingival plaque were pelleted at 1,300 × g for 10 min and washed with TE buffer. Gram-negative strains were lysed with 10% SDS and Proteinase K (20 mg/mL) while Gram-positive strains were lysed with 15 mg/mL lysozyme (Sigma) and 5 mg/mL achromopeptidase (Sigma). Following, cells were sonicated and incubated at 37°C for 1 h. DNA was isolated and purified as described previously by Smith et al. (33). DNA probes were generated for each strain by labeling with digoxigenin (Roche, Basel, Switzerland) (34). Next, the DNA content of the subgingival plaque samples was amplified using multiple displacement amplification (35). Briefly, subgingival DNA templates were mixed with random hexamer primers in 50 mM Tris-HCl (pH 8.2) and 0.5 mM EDTA. The solution was heat denatured at 95°C for 3 min in a thermocycler. DNA polymerase with dNTPs was then added and incubated at 30°C for 2 h. The subgingival plaque samples along with 1 and 10 ng of known DNA standards were analyzed by using checkerboard DNA-DNA hybridization as previously described by Socransky et al. (36). In brief, samples were boiled for 10 min in 1 mL of TE buffer and were loaded on a nylon membrane (Roche, Basel, Switzerland) using a Minislot 30 apparatus (Immunetics, Cambridge, MA, USA). Then cross-linked by ultraviolet light using UV-crosslinker (Stratalinker 1800, La Jolla, CA, USA). Probes were selected according to their importance in periodontal diseases (36), and were subsequently bound perpendicular to the samples on the membrane and were detected using anti-digoxigenin antibody conjugated with alkaline phosphatase and a chemifluorescent substrate. Signal intensities of the plaque samples were measured using a Typhoon TRIO+ Scanner (GE Healthcare). Finally, absolute counts were generated by comparing with signal intensities of standards using Phoretix Array Software (TotalLab) (35, 36).

### Principal Coordinate Analysis

The differences between control and diseased groups were investigated by applying Principal Coordinates Analysis (PCoA) on the binary Jaccard distance metric using ClusterApp tool (available at http://dorresteinappshub.ucsd.edu:3838/clusterMetaboApp0.9.1/) and visualized via EMPeror software (37). Pairwise permutation MANOVA (ADONIS within RVAideMemoire package in R) with 999 permutations were used to test significant differences between sample groups.

### Phylogenetic Analysis

The 16S rRNA gene sequences were downloaded from the Human Oral Microbiome Database (HOMD; version 14.51, January 2017), and were aligned using ClustalW software within Molecular Evolutionary Genetics Analysis (MEGA) software version 7 (38). The phylogenetic tree was plotted by applying the Neighbor-Joining method using p-distance model and by implementing 1,000 bootstrap replications. Species abundance data and the phylogenetic tree were jointly visualized further using the interactive tree of life (iTOL) version 4 (available at http://itol.embl.de/) (39).

### Cytokines Measurement

Whole blood serum collected from the subjects were immediately stored at −80°C. ProcartaPlex Immunoassays include a platform which allows for detection up to 100 proteins simultaneously through magnetic beads. A panel of 20 inflammatory cytokines were measured in duplicate by ProcartaPlex Human Inflammation Panel 20-plex (ThermoFisher Scientific, San Diego, USA) on a Luminex 200 System (Luminex Corporation, Texas, USA) as previously described (40) and adapted according to the manufacturer's specifications. Positive and negative controls were assayed on each plate. The cytokines measured were macrophage inflammatory protein 1 alpha (MIP-1-α), interleukin (IL)-1 beta (β), IL-4, IL-6, IL-8, IL-10, IL-12p70, IL-13, IL-17A, IL-1α, interferon gamma-Induced Protein 10 (IP-10), interferon gamma (IFN-γ), granulocyte-macrophage-colony-stimulating factor (GM-CSF), tumor necrosis factor alpha (TNF-α), monocyte/macrophage inflammatory protein-1 beta (MIP-1β), interferon alpha (IFN-α), monocyte chemoattractant protein 1 (MCP-1), Platelet-selectin (P-selectin), soluble intercellular adhesion molecule-1 (sICAM-1), Endothelial-selectin (E-selectin). Cytokine abundance levels were reported in pg/mL by the assay system. Quality control of the machine-generated raw data was performed using xPONENT 4.2 software as per manufacturer's guidelines (Affymetrix eBioscience, San Diego, USA).

### Statistical Analyses

Statistical analyses were applied to the microbial and cytokine abundance data to detect any features that are significantly different between diseased and control groups. Rank-based tests were employed as a robust non-parametric group comparison tool for those non-normally distributed microbiota and cytokine data. Using Wilcoxon rank-sum test, we further analyzed differences between groups i.e., control vs. SLE-I, control vs. SLE-A and SLE-I vs. SLE-A. For significance testing, FDR-corrected (Benjamini-Hochberg) *p*-values were set at 5% threshold. For binary abundances we dichotomized the relative abundances data into presence (i.e., abundance > 0) and absence (i.e., abundance = 0) binary values. We used a logistic regression model to analyze the relationship between the binary abundance data and two clinically important categorical covariates: SLE group and periodontal condition (41). For the disease group, we have three levels (control, SLE-A, and SLE-I); for the periodontal condition, we have two levels (control vs. periodontitis).

Potential associations between microbial species and cytokine abundances were evaluated by Spearman's rank based correlations. Spearman's correlation coefficients were calculated using R package psych, and significant correlations were plotted with R package corrplot. Pairwise correlations were computed separately for control and SLE samples. To avoid false positives, we selected correlations with adjusted *p* < 0.05. For visual simplicity, we showed only the significant correlations. Topological parameters were analyzed by importing the pairwise correlations using network analyzer algorithm within Cytoscape, 3.7.1. Hierarchical clustering was applied on the relative abundance data of species in each sample based on Spearman rank correlation and the heatmap was generated using ggplots package in R.

## Results

### Clinical Findings of the Cohort

To investigate the role of the periodontal microbiota-induced inflammation on SLE subjects, whole blood, crevicular fluids, and plaque were collected from subjects enrolled in the study. General characteristics of patients are shown in Table 1. A total of 91 subgingival samples were analyzed from women aged 18–65. SLE activity was determined by SLEDAI > 2 (43). Of 91 subjects, 31 individuals were controls, 29 were SLE-I and 31 were SLE-A. The average SLE disease activity index (SLEDAI) for SLE-A subjects significantly increased (7.29 ± 4.31), while SLE-I showed a lower SLEDAI (1.07 ± 0.99). Patients with SLE-I are considered chronic cases, had a longer duration of the disease and aged significantly older (37.69 ± 8.97 years; *p* = 0.045) than SLE-A patients which are considered acute cases, and were younger (32.58 ± 8.9 years) than controls (37.42 ± 8.72 years), as well. SLE-I individuals showed increased clinical attachment loss (CAL) and worsen periodontal clinical outcomes (8.31 ± 9.438) than SLE-I and healthy controls, but with no significant differences between groups, except for bleeding on probing, which was significantly reduced in both SLE-A and SLE-I compared to controls. All periodontal patients presented localized, chronic periodontitis, according to the classification proposed (7), with no differences between groups in the severity or extension of the lesions. The majority of oral health indices including teeth number, percentage of bleeding sites (BOP), plaque index, and probing depth were similar between SLE groups. Ethnicity was equally distributed among the three groups of the studied population. More details see Supplementary Table 4.

**Table 1:** Demographic and clinical features of SLE and control subjects.

		**Control^a^**	**SLE-Inactive^a^**	**SLE-Active^a^**	*p*-value^*^
**DEMOGRAPHICS**					
Total subjects		31 (34.06%)	29 (31.86%)	31 (34.06%)	
Gender			100% female subjects		
Age		37.42 ± 8.72	37.69 ± 8.97	32.58 ± 8.92	0.0448^*^
Ethnicity	Black	8 (25.81%)	3 (10.34%)	7 (22.58%)	
	Mixed	16 (51.61%)	18 (62.07%)	20 (64.52%)	0.3929 ^b^
	White	7 (22.58%)	8 (27.59%)	4 (12.90%)	
**ORAL HEALTH- PERIODONTAL PARAMETERS**					
Teeth		23.32 ± 4.56	23.72 ± 5.404	24.06 ± 4.131	0.8251
Bleeding points		23.77 ± 20.36	14.66 ± 12.32	15.84 ± 21.61	0.1225
BOP (%)		17.33 ± 14.90	11.38 ± 11.92	11.19 ± 14.62	0.1513
Stainned sites		55.58 ± 21.68	55.34 ± 20.45	52.58 ± 20.06	0.8201
Plaque index		60.05 ± 20.90	61.69 ± 24.91	56.65 ± 23.62	0.6900
PD (mean)		2.354 ± 0.35	2.41 ± 0.27	2.39 ± 0.40	0.8375
PD ≥4 mm (no. of teeth)		10.52 ± 12.94	9.79 ± 7.13	10.16 ± 8.65	0.9611
CAL (mean)		0.61 ± 0.33	1.00 ± 1.16	0.72 ± 0.53	0.1157
CAL ≥4 mm (no. of teeth)		5.65 ± 4.48	8.31 ± 9.44	7.55 ± 9.11	0.4104
Classification of PD (42)	Non-periodontitis control	8 (25.81%)	9 (31.03%)	13 (41.94%)	0.38^b^
	Periodontitis	23 (74.19%)	20 (68.96%)	18 (58.06%)	
**SYSTEMIC HEALTH- SLE PARAMETERS**					
Time with SLE (years)		0	11.90 ± 6.91	6.68 ± 4.06	<0.0001^****^
SLEDAI		0	1.07 ± 0.99	7.29 ± 4.31	<0.0001^****^
SLEDAI subgroups^c^	SLEDAI (0)	31 (100%)	13 (44.83%)	0	
	SLEDAI (1–5)	0	16 (55.17%)	14 (45.16%)	
	SLEDAI (6–8)	0	0	9 (29.03%)	
	SLEDAI (9–19)	0	0	7 (22.58%)	
	SLEDAI (>20)	0	0	1 (3.23%)	

a Values represent mean ± SD or percentage (%);

* *p-values for group comparisons were calculated by one-way ANOVA for numerical features*.

b *Chi-square test for categorical features*.

c *n (% within group)*.

*SLE, systemic lupus erythematosus; CP, chronic periodontitis; BOP, bleeding on probing; PD, probing depth; CAL, amount of clinical attachment loss; SLEDAI, systemic lupus erythematosus disease activity index*.

### Inflammatory Cytokines Upregulation in SLE

**Figure 1:**
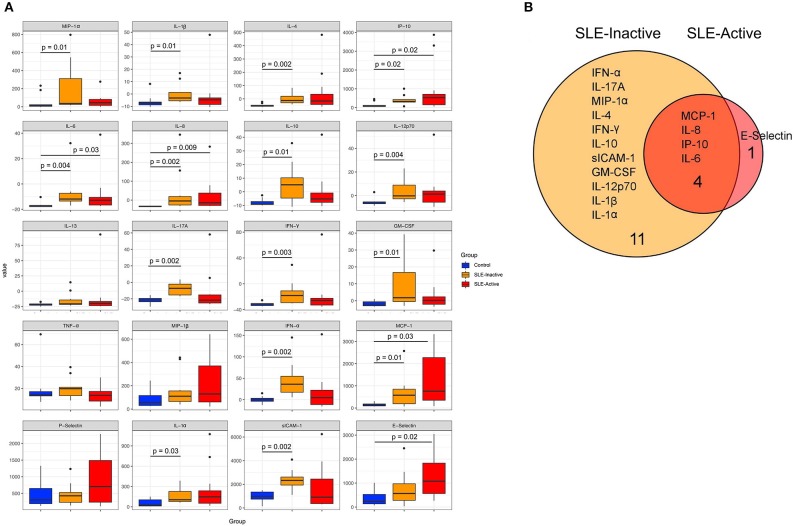
Human inflammation cytokines among control and SLE groups. **(A)** Variation in host cytokine response across control subjects and subjects with SLE-active and SLE-inactive conditions. The y-axis specifies the cytokine levels measured by a multiplex immuno-bead assay. Horizontal bar signifies the mean value for each group. Significance was evaluated by non-parametric Wilcoxon rank-sum test with Benjamini & Hochberg adjusted *p*-values. **(B)** A Venn diagram illustrating overlapping/non-overlapping cytokines in SLE-inactive and SLE-active conditions when compared to their respective controls. Venn diagrams are produced using VennDIS software (*n* = 91; *p*-values for two group comparisons are provided in Table 2).

Various inflammatory cytokines have been involved in regulating disease activity and in organ pathologies of patients diagnosed with SLE (21, 22). In order to identify specific markers responsible for systemic inflammation in SLE patients, we investigated whole blood serum concentrations of pro and anti-inflammatory cytokines from SLE groups compared to healthy controls. We measured proinflammatory levels of MIP-1-α, IL-1β, IP-10, IL-6, IL-8, IL-12p70, IL-17A, IFN-γ, GM-CSF, TNF-α, MIP-1β, IFN-α, MCP-1, P-Selectin, IL-1α, sICAM-1, E-selectin, and anti-inflammatory cytokines IL-4, IL-10, IL-13. Our results demonstrated that twenty cytokine concentration comparisons were upregulated in SLE subjects when compared to healthy controls (Figure 1A, Supplementary Table 1), with sixteen unique profiles among SLE groups (Figure 1B). Proinflammatory cytokine that were upregulated included SLE-I only (IFN-α, IL-17A, MIP-1-α, IFN-γ, IL-10, siCAM-1, GM-CSF, IL-12p70, IL1β, IL-1α), and E-selectin was unique to SLE-A only (Figure 1B). SLE-I sera also demonstrated anti-inflammatory increase by upregulation of IL-4 and IL-10, this was not evident on SLE-A. Among the significant differences from SLE, inactive and active groups, there were four molecules that overlapped (MCP-1, IL-8, IP-10, IL-6). These results suggest that increased low-grade systemic inflammation was observed in SLE patients when compared to healthy controls.

### Subgingival Microbial Compositions in SLE

**Figure 2:**
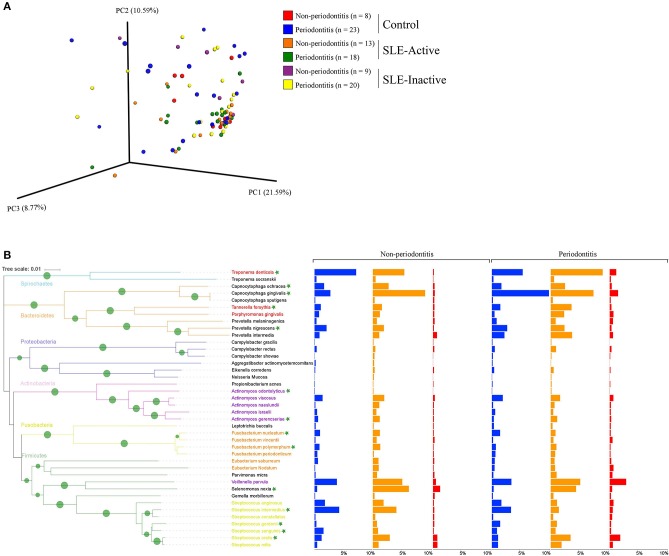
Bacterial composition in control and SLE individuals. **(A)** Principal coordinates analysis (PCoA) of the bacterial community between control and SLE individuals. The principal coordinates were calculated using Jaccard distance metric based on a binary matrix representing the presence/absence of the microbes across control and SLE subjects. Distance among samples in control vs. inactive and active SLE conditions are visualized through EMPeror tool. The significance of separation between groups was tested by applying PERMANOVA test on the principal coordinates. **(B)** Prevalence of disease associated with bacterial species in non-periodontitis and periodontitis individuals. Bar graphs from left to right indicate differences in relative abundance of microbial species in control (in blue), SLE-inactive (in orange), and SLE-active individuals (in red). Branches in the tree are colored according to the phylum and periodontal pathogens are colored according to the red, purple, orange, and yellow bacterial complex designations. Nodes with bootstraps higher than 80% are displayed with the tree. Indicator species with significant differences in abundances between periodontitis and non-periodontitis subjects are indicated with asterisks.

To visualize variations in the composition of subgingival biofilm-associated bacterial species across samples within SLE with or without periodontitis from controls (*n* = 31), SLE-inactive (*n* = 28), and SLE-active (*n* = 31) groups; we performed a collection of samples by probing the gingival site with PerioPaper strips, following elution. After hybridization with specific periodontal pathogen probes, DNA-checkerboard revealed pathogen signatures and abundances. A principal coordinate analysis (PCoA) on the Jaccard distances was generated taking into consideration of periodontal status stratification (Figure 2A). These visual patterns were confirmed by beta-diversity analysis using pairwise permutation Multivariate analysis of variance (MANOVA), revealing significant differences in bacterial species in the SLE-active group without periodontitis and SLE-inactive group with periodontitis compared to control group with periodontitis (*p* = 0.045; Table 2). Lastly, no differences were found between SLE-inactive groups and healthy controls (Supplementary Table 2). Taken together, we found significant variations in the microbiota composition in the SLE when compared to controls, with increased differences when SLE groups were stratified into SLE-A and SLE-I.

**Table 2:** Beta-diversity pairwise comparisons between sample groups.

**Factors**	**Groups**	**Non-periodontitis**			**Periodontitis**	
		**Healthy**	**SLE active**	**SLE inactive**	**Healthy**	**SLE active**
Non-periodontitis	SLE Active	0.253	–	–	–	–
	SLE inactive	0.596	0.293	–	–	–
Periodontitis	Healthy	0.911	0.045^*^	0.606	–	–
	SLE Active	0.38	0.701	0.559	0.253	–
	SLE inactive	0.253	0.329	0.617	0.045^*^	0.559

* *Statistical significance*.

### Microbial Signatures Associated With SLE and Periodontal Phenotypes

**Figure 3:**
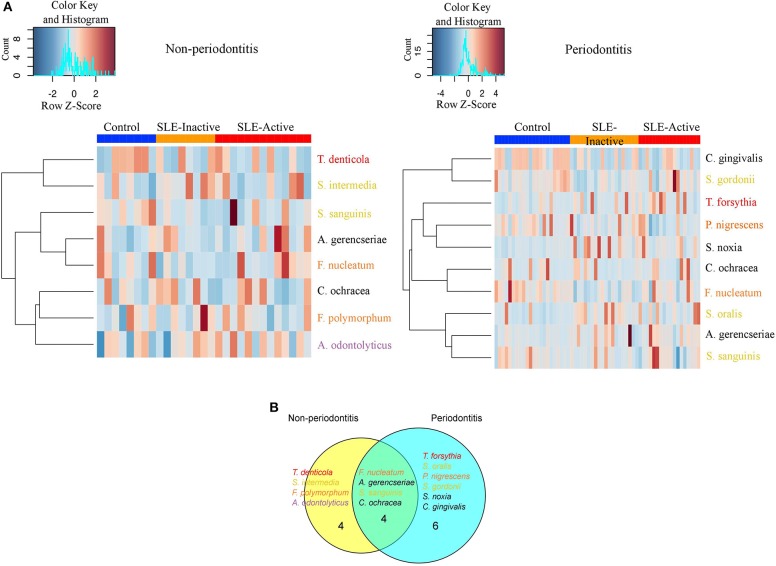
Non-periodontitis and periodontitis associated microbial species in SLE. **(A)** Heatmap illustrating the clustering of 14 bacterial species with significant changes in abundances across control and SLE subjects. Subjects are shown in columns, while bacterial species shown in rows are colored by bacterial complex designations. A red and blue color affected by the row z-score signifies a higher and lower abundance of bacterial species, respectively. Significance was evaluated by Wilcoxon rank-sum test using Benjamini & Hochberg adjusted *p* < 0.05. **(B)** A Venn diagram illustrating shared and uniquely perturbed bacterial species across non-periodontitis and periodontitis individuals with SLE conditions. In each group, subjects were compared to their respective controls. The number of species differentially abundant in the comparison group. Venn diagrams are produced using VennDIS software.

We next sought to understand the underlying microbial associations among SLE subjects and their clinical phenotypes. To investigate the subgingival plaque bacterial composition, we thus applied Wilcoxon rank-sum test on two datasets related to SLE phenotypes. The first dataset was based on relative abundance and the second dataset was based on logistic regression model binary abundances (presence/absence) to evaluate prevalence. Based on the relative abundance profiles, and the hierarchical clustering using Spearman's rank correlation coefficients, we identified two distinct groups of bacterial species in SLE individuals with and without periodontitis when compared to their respective controls (Figures 2B, 3A). Intriguingly, these comprised of species representatives of pathogenic groups previously classified in clusters including the red complex (36) (*Treponema denticola and Tannerella forsythia*), purple complex (*Actinomyces odontolyticus, A. gerencseriae)*, orange complex *(Fusobacterium nucleatum, F. polymorphum*), and yellow complex (*Streptococcus intermedius, S. gordonii, S. sanguinis, S. oralis*). Additionally, *Capnocytophaga ochraceae, C. gingivalis, Prevotella nigrescens*, and *Selenomonas noxia* showed significant differences in their relative abundances among SLE and control subjects.

When further stratifying the SLE comparisons according to periodontal status, the results showed that systemic comparisons were significantly different according to periodontal status (Supplementary Table 2). A total of 24 bacterial species had significant differential abundances among SLE and control subjects, with nine in the non-periodontitis and 15 in the periodontitis groups (Figure 3A, and *p*-values listed in Supplementary Table 2). In non-periodontitis subjects, SLE-I vs. healthy controls showed no significant microbial changes, demonstrating similar microbial abundances among these groups. In contrast, SLE-A vs. healthy controls showed five significant microbial differences including *C. ochracea, F. nucleatum, S. sanguinis, A. gerencseriae, T. denticola*. When comparing SLE-A vs. SLE-I subjects, four signatures were significant including, *C. ochracea, S. intermedia, A. odontolyticus, F. polymorphum*. Whereas, in the periodontitis subjects, SLE-I vs. healthy controls showed three significant microbial changes *A. gerencseriae, S. oralis, S. noxia*. An increase to seven significant microbial abundance changes were observed on SLE-A vs. healthy controls comparison, including, *C. ochracea, F. nucleatum, P. nigrescens, S. sanguinis, C. gingivalis, T. forsythia, S. gordonii*. Finally, when comparing SLE-A vs. SLE-I subjects, five signatures were significantly different including, *C. ochracea, P. nigrescens, A. gerencseriae, S. oralis*, and *T. forsythia*. While the long-term chronically inflamed SLE-I subjects showed higher abundances of periodontal pathogens when compared to SLE-A, SLE-I only showed significance difference to healthy controls in periodontitis groups. Overall the results suggest that the presence of periodontitis drove most of significant microbial differences among the SLE systemic status comparisons (SLE-I or SLE-A vs. healthy controls, and SLE-A vs. SLE-I).

We further characterized the periodontal pathogens in sites from SLE-I vs. SLE-A individuals. Proportions of *C. gingivalis, S. gordonii, P. nigrescens, C. ochracea, F. nucleatum*, and S. sanguinis were significantly reduced on SLE subjects (Figures 2B, 3A, Supplementary Table 2). Based on our analysis the red complex pathogen, *T. forsythia*, was enriched in periodontitis subjects from SLE-active, but not in their healthy counterpart. In addition to these changes, we found *S. noxia, S. oralis and A. gerencseriae* at higher abundance in the SLE-I group compared with control individuals with periodontitis (Figures 2B, 3A). *A. gerencseriae, S. oralis, C. ochracea, P. nigrescens, and T. forsythia* were also found to be significantly different between SLE-I and SLE-A groups. These results suggest unique pathogen signatures associated with SLE-A phenotype and that commensals are associated with better systemic and periodontal health outcomes. A more stringent evaluation of bacterial abundance profiles via binary abundances revealed significant differences in the presence/absence of *C. ochracea, C. gingivalis, P. nigrescens, T. forsythia, F. nucleatum, S. gordonii, and S. sanguinis* in periodontal sites of the SLE-active group compared with the control group (Supplementary Table 3).

Unique and overlapping microbial species were identified among periodontitis vs. non-periodontitis groups (Figure 3B). Among fourteen unique microbial species, four bacterial species overlapped among non-periodontitis and periodontitis individuals (*F. nucleatum, A. gerencseriae, S. sanguinis, and C. ochracea*). While four unique bacterial species (*T. denticola, S. intermedia, F. polymorphum, and A. odontolyticus)* were significantly abundant only in non-periodontitis subjects, six species were unique in periodontitis subjects (*T. forsythia, S. oralis, P. nigrescence, S. gordonii, S. noxia, and C. gingivalis;* Figure 3B). Together these results suggest periodontal bacterial diversity were lower in SLE patients with enrichment of specific periodontal pathogens dominating the microbial environment.

### Bacterial Species and Cytokine Co-occurrences

**Figure 4:**
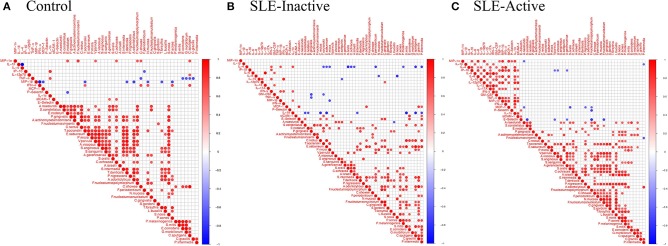
Bacterial species and cytokine co-occurrences. Spearman rank based pairwise correlation analysis between bacterial species and inflammatory cytokines **(A)** in control, **(B)** SLE-inactive, and **(C)** SLE-active subjects. Only significant correlations (*p* < *0.05*) were plotted. The size of the spheres represents *p*-value. While strong correlations are shown by large circles, weak correlations are shown by small circles. The color of the circle denotes the strength of the correlation. Perfect positive correlation (with correlation coefficient 1) are indicated in dark red, whereas perfect inverse correlation (with correlation coefficient −1) are colored in dark blue.

Periodontitis is caused by the complex interplay between subgingival microbiota composition and the host immune response. To unravel functional insights, we investigated microbe-inflammatory cytokines correlations in periodontal and SLE phenotypes. The analysis showed associations between bacterial abundance and host cytokine levels in control, SLE-inactive and SLE-active individuals by Spearman's rank-based correlations (Figures 4A–C, Supplementary Figure 1). Among the relationships found, a total number of 77 connecting edges of cytokine-cytokine, microbe-microbe, and microbe-cytokine was evident on SLE-A when compared to controls and SLE-I (Figures 4B,C). The controls showed negative values for most cytokines thus excluded from correlation plots. The majority of differentially abundant bacterial species perturbed cytokine levels, except *S. mitis*, which had more connectivity with other bacterial species in SLE individuals vs. healthy controls.

In control individuals, the presence of pathogenic species, such as *T. denticola, A. odontolyticus*, and *T. forsythia*, was found to be negatively correlated with MIP-1β levels (Figure 4A, Supplementary Figure 1A); *C. ochracea* abundance positively correlated with MIP-1-α, sICAM-1 while *F. polymorphum* positively correlated with MIP-1-α levels. *A. gerencseriae* is positively correlated with E-selectin levels. In SLE-inactive individuals, we found co-occurrence of *F. nucleatum* with E-selectin levels (Figure 4B, Supplementary Figure 1B). In contrast, the presence of health-related *S. sanguinis* inversely correlated with IP-10, while *A. odontolyticus* and *C. gingivalis* were positively associated with MCP-1 and IL12p70 respectively. *C. ochracea* inversely correlated with IL-4. *S. intermedia* inversely correlated with IL-1α. *S. oralis* negatively correlated with IL-4. *S. noxia* negatively correlated with IL-8. In SLE-active individuals, *A. gerencseriae* is found to vary inversely with IP-10. *F. polymorphum* negatively correlated with MCP-1, E-selectin, and P-selectin*. S. oralis* and *P. nigrescens* showed negative associations with MCP-1 (Figure 4C, Supplementary Figure 1C).

The degree of connectivity (i.e., number of connecting edges to neighboring nodes) of *F. nucleatum* with other bacterial species and cytokines was higher in individuals with SLE when compared to control individuals. A*. gerencseriae, C. ochracea*, and *T. forsythia* showed higher connectivity in control and in SLE-A individuals while connectivity of *S. oralis* and *P. nigrescens* were elevated in SLE-A subjects. *S. noxia* was higher in the control group while *C. gingivalis* had no connectivity in control individuals (Supplementary Figure 2). These findings suggest that subgingival bacterial species associated with SLE govern systemic host cytokine patterns impacting overall health.

## Discussion

At the center of autoimmune pathogenesis, this study provides evidence that inflammatory response specific to oral bacteria controls not only the disease severity, but also the magnitude of the SLE disease (44). The data also indicates that periodontal levels are also associated with low-grade systemic inflammation. Oral microbes, including subgingival bacteria, are involved in homeostasis and maintenance of health, and also in the initiation and progression of chronic periodontitis (CP) which leads to systemic inflammation such as SLE (45–51). Dual relationships among systemic and oral diseases play a role in the pathogenesis, and low-grade inflammation modulating the biological compartments (19), and here we assayed how the microbial species influence specific systemic cytokines. Evidence of the exact repertoire of subgingival pathogens influencing specific lupus phenotypes has been scarcer in the context of oral and systemic disease. We explored two distinct populations, healthy and SLE positive patients to determine these correlations. Our clinical cross-sectional study is unique because we have stratified chronic SLE patients, which are usually under treatment (SLE-inactive), and a more acute group, and recently diagnosed SLE subjects (SLE-active). We have also carefully stratified the subjects in periodontally compromised vs. healthy controls. Our results suggest that chronic and SLE-inactive subjects are positively associated with severe periodontitis states. Among healthy and SLE groups, age was not significantly different, but when stratifying SLE active and inactive groups, younger subjects were found significantly present on SLE-A when compared to SLE-I (Table 1). This was expected due to time of disease since diagnosis (52). Patients with SLE in general showed clear molecular patterns related to chronic inflammation and immune response to oral pathogens when compared to healthy controls.

A more distinct host cytokine dysregulation was associated with SLE subjects when compared to healthy controls. The data presented herein show no evidence that SLE-active subjects had higher serum concentrations of pro- and anti-inflammatory cytokine. But, both SLE groups showed higher expressions than healthy control. Interestingly IL-10 which is a anti-inflammatory cytokine response was found increased in SLE-I inactive when compared to active and controls (*p* = 0.01, Figure 1A). Our results showed significantly increased levels of IFN-α, IL-17A, MIP-1-α, IL-4, IFN-γ, and IL-10 in SLE-inactive individuals. sICAM-1, GM-CSF, IL-12p70, IL-1β, IL-1α in serum were unique of SLE-inactive subjects, while four cytokines were significantly increased, with one unique profile including E-selectin of SLE-A subjects (Figure 1B). A high number of cytokine perturbations in SLE-I individuals can be associated with diverse clinical manifestations, activity and the severity of the disease. As mediators of inflammation, cytokine production feeds forward cell-cell and cell-tissue communications guiding health and disease phenotypes and organ disruptions. In the present study we considered IL-4 and IL-10 as anti-inflammatory cytokines due to their specific actions on innate immune cells, and here we have shown that proinflammatory cytokines MCP-1, IL-8, IP-10, and IL-6 were significantly elevated in SLE-I and SLE-A patients in comparison to non-SLE subjects (Figure 1A). Of this proinflammatory cytokines, IL-6, IL-17, TNF-α, and IFN-α suppression have been reported as therapeutic targets for clinical management of lupus and other chronic inflammatory diseases (2, 53–55).

Systemic and local periodontal inflammation can impact subgingival bacterial compositions, which can in turn further enhance systemic inflammation, leading to tissue loss, especially in subjects with SLE condition (53). The bacterial abundances of 40-selected subgingival species were evaluated through checkerboard and we found that two distinct groups of subgingival bacteria were present in SLE individuals, in fact, when stratifying for the presence of absence of periodontitis (Figure 3A). *S. sanguinis* and *C. ochracea* are known to be prevalent in healthy subgingival sites (56–58). In line with previous studies, proportions of *T. denticola, S. sanguinis*, and *C. ochracea* were significantly higher in periodontally healthy individuals than in individuals with SLE. *A. gerencseriae* and *F. nucleatum* were more evidently abundant in SLE individuals without periodontitis. We found a higher abundance of *S. noxia, S. oralis*, and *A. gerencseriae* in SLE subjects with periodontitis. On the other hand, we found significant depletion of *C. gingivalis, S. gordonii, P. nigrescens, C. ochracea, F. nucleatum*, and *S. sanguinis*, which were previously found to be associated with SLE pathogenesis (Figure 3A, Supplementary Tables 2, 3). It is noteworthy that SLE-I microbial compositions were not different from healthy controls in the non-periodontitis group, indicating that they are similar in periodontally healthy subjects (Supplementary Table 2). On the contrary, we show that SLE-A microbial positions maintains differences in both periodontitis and non-periodontitis groups, and that one form of inflammation (local or systemic) control these differences associated with SLE systemic inflammatory profiles.

Co-occurrence of subgingival bacteria and cytokines also differed among controls, SLE-I and SLE-A subjects. Co-occurrence among cytokines was more evident (77 positive connecting edges) in the SLE states, suggesting positive association among cytokines are an important driver of SLE pathogenesis (Figures 4A–C). We did not find any correlations between bacteria and increased levels of IL-6 and IL-17. Despite IFN-α and TNF-α co-occurred with certain subgingival bacteria, these bacteria were not differentially abundant. Beyond cytokine to microbe interactions, SLE-A showed higher cytokine-cytokine co-occurrences. IL-1β, IL-8, and IL-12 are associated with increased innate and adaptive immune response including T-cell. IL-1β with high inflammatory properties has been shown to increase in SLE condition with systemic manifestations, especially in the inflammasome activation associated with inflammatory responses to extracellular pathogens. IL-8 has been shown to induce neutrophil recruitment, especially during early stages of SLE. Qiu et al. reported high levels of IL-12 family-related cytokines in triggering the production of anti-double-stranded DNA antibodies (59).

Functional inflammation data at the host and microbiota levels are necessary to make assumptions on ecological systems from the oral cavity and its influences on systemic disease phenotypes. The data presented here generate additional questions about the extent to which detrimental and protective microbiome-related features are innately acquired and significantly modulated by the host systemic conditions. Overall the results offer a new perspective on the host-microbial interactions in lupus disease and periodontal pathogenic environments. Consequently, these results favor the hypothesis of oral-systemic inflammation linking both conditions. Moreover, the realization that there remain high anti-inflammatory profiles on subjects with inactive disease when compared to active, offer a mechanistic hypothesis to the lack of disease comorbidities of this suppressed condition, but the host is still able to produce low-grade inflammation systemically. In future studies using next generation sequencing strategies of highly diverse SLE subjects, it will be possible to map the microbial ecology of multiple sites of the human body, including oral sites. As the science of host-microbes is advancing, highly individualized, rather than simple binary distinctions of healthy and disease or single-microbe etiology across populations, might be found (60).

The results presented here show that oral-systemic pathogenic burden is evident in lupus subjects. These data are also consistent with complex ecological interactions among multiple human chronic diseases (61). Among the relationships found, co-occurrence within the cytokines was evident on SLE-A group (a total number of 77 connecting edges, Figure 4C) when compared to controls and SLE-inactive states (Figures 4A,B), suggesting positive relationships of proinflammatory cytokines as important drivers of SLE pathogenesis. Currently, there is no cure for SLE manifestations, and administration of immunosuppressive drugs are aimed at suppressing immune signals chronically, and removal of the etiological factors nor disruption of the human microbiome. It is possible, in the future, that by controlling oral microbiome-induced inflammation, the systemic low-grade inflammation burden will be reduced, preventing future emergence of comorbidities and disease clusters, emphasizing the need of adding periodontal diagnosis and treatment as part of management of systemic diseases. Thus, periodontal and oral diseases are biological sources of complex ecosystem influencing systemic inflammation. These findings, along with future longitudinal studies, could provide improved resolution on how microbial and immune features interact to modify disease outcomes on selected individuals and populations. This strategy is key to trace how health and disease-associated oral biomarkers are spatially and temporally regulated in the context of chronic human diseases.

## Data Availability Statement

All datasets generated for this study are included in the article/Supplementary Material.

## Ethics Statement

This research project was approved by the Bauru Dental School-USP Ethics Committee of Research (protocol # 111.718) and by the Ethics Committee of Research at the Forsyth Institute (IRB #037). The patients/participants provided their written informed consent to participate in this study.

## Author Contributions

AS and MF conceived and designed the experiments. LP, LN, DN, and TY performed the experiments. YZ, WL, and GA analyzed the data. AS, MF, and KN contributed reagents, materials, and analysis tools. MF, AS, and SC wrote the paper.

### Conflict of Interest

The authors declare that the research was conducted in the absence of any commercial or financial relationships that could be construed as a potential conflict of interest.

## Supplementary Material

Supplementary Figure 1 Significant Spearman correlations among bacteria and host cytokines in **(A)** control, **(B)** SLE-inactive, and **(C)** SLE-active states. The size of the node (circles) in SLE-inactive and the SLE-active group represents a log_2_ fold change of abundances in comparison to control subjects. Increase in the size of the node signifies lower expression in control when compared to SLE subjects. Edges in red represent negative correlations, gray represent positive correlations.

Supplementary Figure 2 Topological analysis of the co-occurrence networks among cytokines and bacterial species. The selected topological parameter illustrates the percentage connectivity of each bacterial species and cytokines within control, SLE-I, and SLE-A networks.

Supplementary Table 1 Comparison of cytokine levels between SLE and control groups. Significance was evaluated by the non-parametric Wilcoxon rank-sum test. Significant *p*-values (Benjamini & Hochberg adjusted) are highlighted in bold.

Supplementary Table 2 Comparison of relative abundances of bacterial species between SLE and control groups with or without periodontitis. Significance was evaluated by the non-parametric Wilcoxon rank-sum test. Significant *p*-values (Benjamini & Hochberg adjusted) are highlighted in bold.

Supplementary Table 3 Comparison of presence/absence of bacterial species between SLE and control groups with or without periodontitis. Significance was evaluated by the logistic regression model. Significant *p*-values (Benjamini & Hochberg adjusted) are highlighted in bold.

Supplementary Table 4 Comprehensive demographics table and clinical comparisons of SLE and control subjects with chronic or non-chronic periodontitis.

## References

[1] Corrêa JD Saraiva AM Queiroz-Junior CM Madeira MFM Duarte PM Teixeira MM. Arthritis-induced alveolar bone loss is associated with changes in the composition of oral microbiota. Anaerobe. (2016) 39:91–6. 10.1016/j.anaerobe.2016.03.006 26996070

[2] Corrêa JD Calderaro DC Ferreira GA Mendonça SMS Fernandes GR Xiao E. Subgingival microbiota dysbiosis in systemic lupus erythematosus: association with periodontal status. Microbiome. (2017) 5:34. 10.1186/s40168-017-0252-z 28320468 PMC5359961

[3] Kobayashi T Ito S Yasuda K Kuroda T Yamamoto K Sugita N The combined genotypes of stimulatory and inhibitory Fcγ receptors associated with systemic lupus erythematosus and periodontitis in Japanese adults. J Periodontol. (2007) 78:467–74. 10.1902/jop.2007.060194 17335370

[4] Scher JU Bretz WA Abramson SB. Periodontal disease and subgingival microbiota as contributors for rheumatoid arthritis pathogenesis: modifiable risk factors? Curr Opin Rheumatol. (2014) 26:424–9. 10.1097/BOR.0000000000000076 24807405 PMC4128331

[5] Socransky SS Haffajee AD Cugini MA Smith C Kent RL. Microbial complexes in subgingival plaque. J Clin Periodontol. (1998) 25:134–44. 10.1111/j.1600-051X.1998.tb02419.x 9495612

[6] Kumar PS Leys EJ Bryk JM Martinez FJ Moeschberger ML Griffen AL. Changes in periodontal health status are associated with bacterial community shifts as assessed by quantitative 16S cloning and sequencing. J Clin Microbiol. (2006) 44:3665–73. 10.1128/JCM.00317-06 17021095 PMC1594761

[7] Armitage GC. Development of a classification system for periodontal diseases and conditions. Ann Periodontol. (1999) 4:1–6. 10.1902/annals.1999.4.1.1 10863370

[8] Rees F Doherty M Grainge MJ Lanyon P Zhang W. The worldwide incidence and prevalence of systemic lupus erythematosus: a systematic review of epidemiological studies. Rheumatology. (2017) 56:1945–61. 10.1093/rheumatology/kex260 28968809

[9] Pons-Estel GJ Alarcón GS Scofield L Reinlib L Cooper GS. Understanding the epidemiology and progression of systemic lupus erythematosus. Semin Arthritis Rheum. (2010) 39:257–68. 10.1016/j.semarthrit.2008.10.007 19136143 PMC2813992

[10] Roper G Lupus Awareness Survey for the Lupus Foundation of America [Executive Summary Report]. Washington, DC. GfK Roper Public Affairs & Corporate Communications (2012).

[11] Wallace D Hahn BH Dubois' Lupus Erythematosus and Related Syndromes. Elsevier Health Sciences (2012). Amsterdam: Elsevier.

[12] Hochberg MC. Updating the American College of Rheumatology revised criteria for the classification of systemic lupus erythematosus. Arthritis Rheum. (1997) 40:1725. 10.1002/art.1780400928 9324032

[13] Agmon-Levin N Mosca M Petri M Shoenfeld Y Systemic lupus erythematosus one disease or many? Autoimmun Rev. (2012) 11:593–5. 10.1016/j.autrev.2011.10.020 22041578

[14] Proal AD Albert PJ Marshall TG. The human microbiome and autoimmunity. Curr Opin Rheumatol. (2013) 25:234–40. 10.1097/BOR.0b013e32835cedbf 23370376

[15] Azzouz D Omarbekova A Heguy A Schwudke D Gisch N Rovin BH. Lupus nephritis is linked to disease-activity associated expansions and immunity to a gut commensal. Ann. Rheum. Dis. (2019) 78:947–56. 10.1136/annrheumdis-2018-214856 30782585 PMC6585303

[16] de Araújo Navas EAF Sato EI Pereira DFA Back-Brito GN Ishikawa JA Jorge AOC. Oral microbial colonization in patients with systemic lupus erythematous: correlation with treatment and disease activity. Lupus. (2012) 21:969–77. 10.1177/0961203312443420 22453994

[17] Mutlu S Richards A Maddison P Scully C. Gingival and periodontal health in systemic lupus erythematosus. Community Dent Oral Epidemiol. (1993) 21:158–61. 10.1111/j.1600-0528.1993.tb00742.x 8348790

[18] Novo E Garcia-MacGregor E Viera N Chaparro N Crozzoli Y. Periodontitis and anti-neutrophil cytoplasmic antibodies in systemic lupus erythematosus and rheumatoid arthritis: a comparative study. J. Periodontol. (1999) 70:185–8. 10.1902/jop.1999.70.2.185 10102556

[19] van der Meulen TA Harmsen HJM Vila AV Kurilshikov A Liefers SC Zhernakova A. Shared gut, but distinct oral microbiota composition in primary Sjögren's syndrome and systemic lupus erythematosus. J Autoimmun. (2019) 97:77–87. 10.1016/j.jaut.2018.10.009 30416033

[20] Figueredo CMS Areas A Sztajnbok FR Miceli V Miranda LA Fischer RG. Higher elastase activity associated with lower IL-18 in GCF from juvenile systemic lupus patients. Oral Health Prevent Dent. (2008) 6:75–81. 18399311

[21] Brugos B Vincze Z Sipka S Szegedi G Zeher M. Serum and urinary cytokine levels of SLE patients. Pharmazie. (2012) 67:411–3. 10.1691/ph.2012.1694 22764573

[22] McCarthy EM Smith S Lee RZ Cunnane G Doran MF Donnelly S. The association of cytokines with disease activity and damage scores in systemic lupus erythematosus patients. Rheumatology. (2014) 53:1586–94. 10.1093/rheumatology/ket428 24706988

[23] Marques CPC Victor EC Franco MM Fernandes JMC Maor Y De Andrade MS. Salivary levels of inflammatory cytokines and their association to periodontal disease in systemic lupus erythematosus patients. A case-control study. Cytokine. (2016) 85:165–70. 10.1016/j.cyto.2016.06.025 27371775

[24] Mackay M Oswald M Sanchez-Guerrero J Lichauco J Aranaw C Kotkin S. Molecular signatures in systemic lupus erythematosus: distinction between disease flare and infection. Lupus Sci Med. (2016). 3:e000159. 10.1136/lupus-2016-000159 27933197 PMC5133406

[25] Petri M Orbai A-M Alarcón GS Gordon C Merrill JT Fortin PR. Derivation and validation of the systemic lupus international collaborating clinics classification criteria for systemic lupus erythematosus. Arthritis Rheum. (2012) 64:2677–86. 10.1002/art.34473 22553077 PMC3409311

[26] Tan EM Cohen AS Fries JF Masi AT McShane DJ Rothfield NF. The 1982 revised criteria for the classification of systemic lupus erythematosus. Arthritis Rheum. (1982) 25:1271–7. 10.1002/art.1780251101 7138600

[27] Bombardier C Gladman DD Urowitz MB Caron D Chang CH Austin A. Derivation of the sledai. A disease activity index for lupus patients, Arthritis Rheum. (1992) 35:630–40. 10.1002/art.1780350606 1599520

[28] Gladman D Ginzler E Goldsmith C Fortin P Liang M Sanchez-Guerrero J. The development and initial validation of the systemic lupus international collaborating clinics/American college of rheumatology damage index for systemic lupus erythematosus. Arthritis Rheum. (1996) 39:363–9. 10.1002/art.1780390303 8607884

[29] Ainamo J Bay I. Problems and proposals for recording gingivitis and plaque. Int Dent J. (1975) 25:229–35. 1058834

[30] O'Leary TJ Drake RB Naylor JE. The plaque control record. J Periodontol. (1972) 43:38–38. 10.1902/jop.1972.43.1.38 4500182

[31] Eke PI Dye BA Wei L Slade GD Thornton-Evans GO Borgnakke WS. Update on prevalence of periodontitis in adults in the United States: NHANES 2009 to 2012. J Periodontol. (2015) 86:611–22. 10.1902/jop.2015.140520 25688694 PMC4460825

[32] Enikov ET Eke E Teaching classical control system course with portable student-owned mechatronic kits. In: ASME 2012 International Mechanical Engineering Congress and Exposition, Education and Globalization; General Topics. Vol. 5. Houston, TX (2012). 10.1115/IMECE2012-86700

[33] Smith GL Socransky SS Smith CM. Rapid method for the purification of DNA from subgingival microorganisms. Oral Microbiol Immunol. (1989) 4:47–51. 10.1111/j.1399-302x.1989.tb00406.x 2628868

[34] Feinberg AP Vogelstein B. A technique for radiolabeling DNA restriction endonuclease fragments to high specific activity. Analyt Biochem. (1983) 132:6–13. 10.1016/0003-2697(83)90418-9 6312838

[35] Brito LCN Sobrinho APR Teles RP Socransky SS Haffajee AD Vieira LQ Microbiologic profile of endodontic infections from HIV– and HIV+ patients using MDA and Checkerboard. Oral Dis. (2012) 18:558–567. 10.1111/j.1601-0825.2012.01908.x 22335194 PMC4148015

[36] Socransky SS Haffajee AD Smith C Martin L Haffajee JA Uzel NG. Use of checkerboard DNA-DNA hybridization to study complex microbial ecosystems. Oral Microbiol Immunol. (2004) 19:352–62. 10.1111/j.1399-302x.2004.00168.x 15491460

[37] Vázquez-Baeza Y Pirrung M Gonzalez A Knight R. EMPeror: a tool for visualizing high-throughput microbial community data. Gigascience. (2013) 2:16. 10.1186/2047-217X-2-16 24280061 PMC4076506

[38] Kumar S Stecher G Tamura K. MEGA7: molecular evolutionary genetics analysis version 7.0 for bigger datasets. Mol Biol Evol. (2016) 33:1870–4. 10.1093/molbev/msw054 27004904 PMC8210823

[39] Letunic I Bork P. Interactive tree of life (iTOL) v3: an online tool for the display and annotation of phylogenetic and other trees. Nucleic Acids Res. (2016) 44:W242–5. 10.1093/nar/gkw290 27095192 PMC4987883

[40] Chalan P Bijzet J van den Berg A Kluiver J Kroesen BJ Boots AMH. Analysis of serum immune markers in seropositive and seronegative rheumatoid arthritis and in high-risk seropositive arthralgia patients. Sci Rep. (2016) 6:26021. 10.1038/srep26021 27189045 PMC4870704

[41] Lee CY Robinson DA Johnson CA Jr Zhang Y Wong J Joshi DJ. A randomized controlled trial of liposomal bupivacaine parasternal intercostal block for sternotomy. Ann Thorac Surg. (2019) 107:128–34. 10.1016/j.athoracsur.2018.06.081 30170012

[42] Eke PI Page RC Wei L Thornton-Evans G Genco RJ. Update of the case definitions for population? Based surveillance of periodontitis. J Periodontol. (2012) 83:1449–54. 10.1902/jop.2012.110664 22420873 PMC6005373

[43] Mosca M Merrill JT Bombardieri S. Assessment of disease activity in systemic lupus erythematosus. In: Systemic Lupus Erythematosus. Philadelphia, PA: Elsevier (2007). p. 19–23.

[44] Fava A Petri M. Systemic lupus erythematosus: diagnosis and clinical management. J Autoimmun. (2019) 96:1–13. 10.1016/j.jaut.2018.11.001 30448290 PMC6310637

[45] Cunningham LL Novak MJ Madsen M Abadi B Ebersole JL. A bidirectional relationship of oral-systemic responses: observations of systemic host responses in patients after full-mouth extractions. Oral Surg Oral Med Oral Pathol Oral Radiol. (2014) 117:435–44. 10.1016/j.oooo.2013.11.502 24630161

[46] Glick M The Oral-systemic Health Connection: A Guide to Patient Care. Batavia, IL: Quintessence Publishing Company (2014).

[47] Kato T Yamazaki K Nakajima M Date Y Kikuchi J Hase K. Oral administration of porphyromonas gingivalis alters the gut microbiome and serum metabolome. mSphere. (2018) 3:e00460–18. 10.1128/mSphere.00460-18 30333180 PMC6193602

[48] Kumar PS. From focal sepsis to periodontal medicine: a century of exploring the role of the oral microbiome in systemic disease. J Physiol. (2017) 595:465–76. 10.1113/JP272427 27426277 PMC5233655

[49] Olsen I Yamazaki K. Can oral bacteria affect the microbiome of the gut? J Oral Microbiol. (2019) 11:1586422. 10.1080/20002297.2019.1586422 30911359 PMC6427756

[50] T.J.C Oral diagnosis of systemic disease. Oral Surg Oral Med Oral Pathol. (1960) 13:896 10.1016/0030-4220(60)90028-1

[51] Valentich MA Cafaro TA Serra HM Oral cavity-associated immune system: what is new? Curr Immunol Rev. (2011) 7:253–63. 10.2174/157339511796196610

[52] Fabbri C Fuller R Bonfá E Guedes LKN D'Alleva PSR Borba EF. Periodontitis treatment improves systemic lupus erythematosus response to immunosuppressive therapy. Clin Rheumatol. (2014) 33:505–9. 10.1007/s10067-013-2473-2 24415114

[53] Jacob N Stohl W. Cytokine disturbances in systemic lupus erythematosus. Arthritis Res Ther. (2011) 13:228. 10.1186/ar3349 21745419 PMC3239336

[54] Nalbandian A Crispín JC Tsokos GC. Interleukin-17 and systemic lupus erythematosus: current concepts. Clin Exp Immunol. (2009) 157:209–15. 10.1111/j.1365-2249.2009.03944.x 19604260 PMC2730846

[55] Tackey E Lipsky PE Illei GG. Rationale for interleukin-6 blockade in systemic lupus erythematosus. Lupus. (2004) 13:339–43. 10.1191/0961203304lu1023oa 15230289 PMC2014821

[56] Galimanas V Hall M Singh N Lynch MD Goldberg M Tenenbaum H. Bacterial community composition of chronic periodontitis and novel oral sampling sites for detecting disease indicators. Microbiome. (2014) 2:32. 10.1186/2049-2618-2-32 25225610 PMC4164120

[57] Wade WG Characterisation of the human oral microbiome. J Oral Biosci. (2013) 55:143–8. 10.1016/j.job.2013.06.001

[58] Wang J Qi J Zhao H He S Zhang Y Wei S. Metagenomic sequencing reveals microbiota and its functional potential associated with periodontal disease. Sci Rep. (2013) 3:1843. 10.1038/srep01843 23673380 PMC3654486

[59] Qiu F Song L Yang N Li X. Glucocorticoid downregulates expression of IL-12 family cytokines in systemic lupus erythematosus patients. Lupus. (2013) 22:1011–6. 10.1177/0961203313498799 23884985

[60] Gomez A Espinoza JL Harkins DM Leong P Saffery R Bockmann M. Host genetic control of the oral microbiome in health and disease. Cell Host Microbe. (2017) 22:269–78.e3. 10.1016/j.chom.2017.08.013 28910633 PMC5733791

[61] Blaser MJ. Who are we? Indigenous microbes and the ecology of human diseases. EMBO Rep. (2006) 7:956–60. 10.1038/sj.embor.7400812 17016449 PMC1618379

