# Supplementary figures and images for: Host-Microbial Interactions in Systemic Lupus Erythematosus and Periodontitis
Source: Front Immunol. 2019 Nov 12;10:2602. doi: 10.3389/fimmu.2019.02602 (PMC6861327; doi:10.3389/fimmu.2019.02602)

Suppl 1.

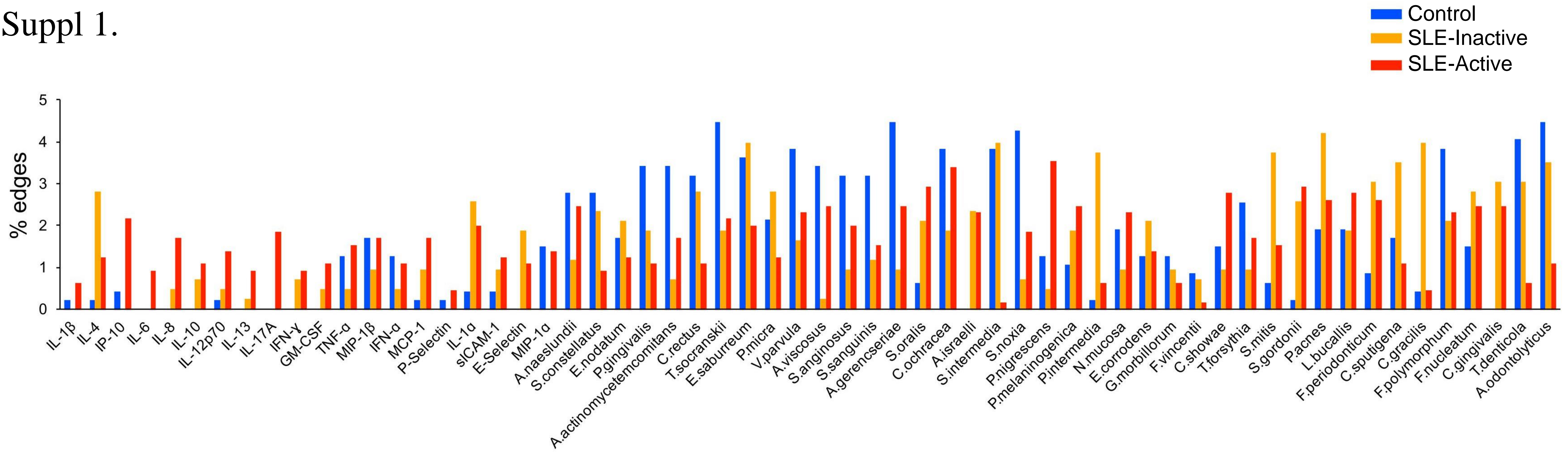

Supplement: Supplementary Figure 1 — Significant Spearman correlations among bacteria and host cytokines in (A) control, (B) SLE-inactive, and (C) SLE-active states. The size of the node (circles) in SLE-inactive and the SLE-active group represents a log2 fold change of abundances in comparison to control subjects. Increase in the size of the node signifies lower expression in control when compared to SLE subjects. Edges in red represent negative correlations, gray represent positive correlations. [file Image_1.pdf]

## Suppl. 2A. Control

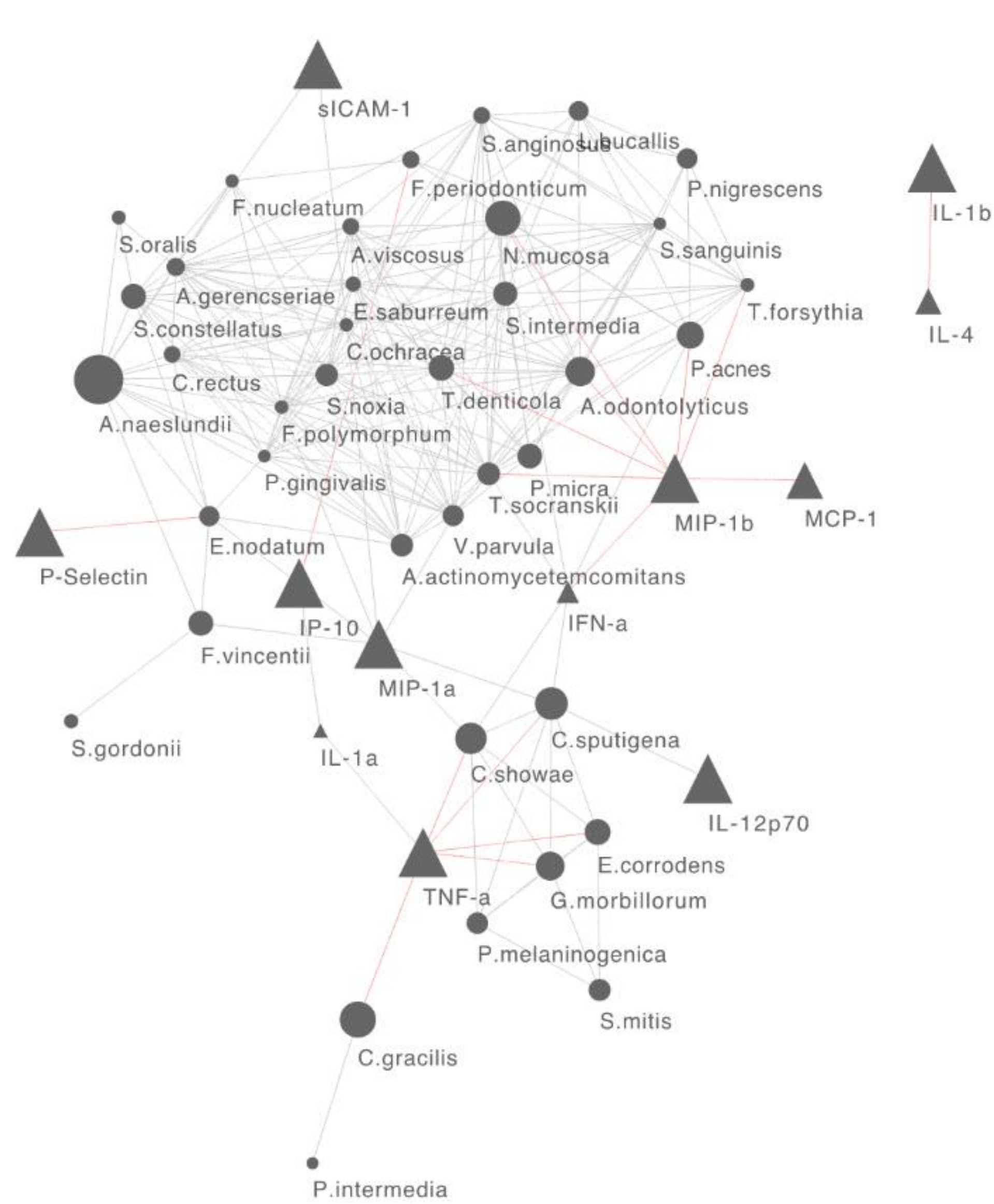

## Suppl. 2B. SLE-Inactive

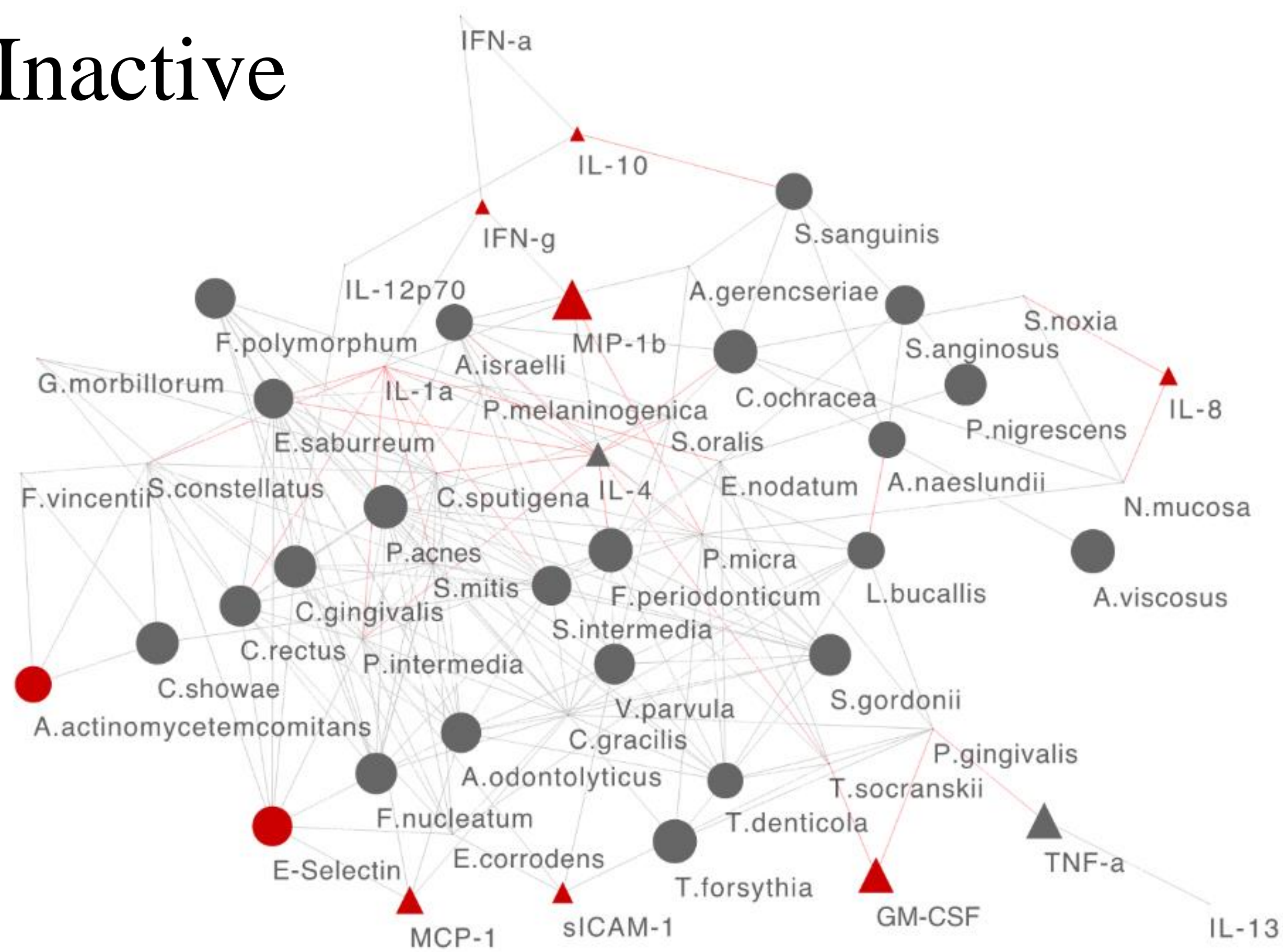

## Suppl. 2C. SLE-Active

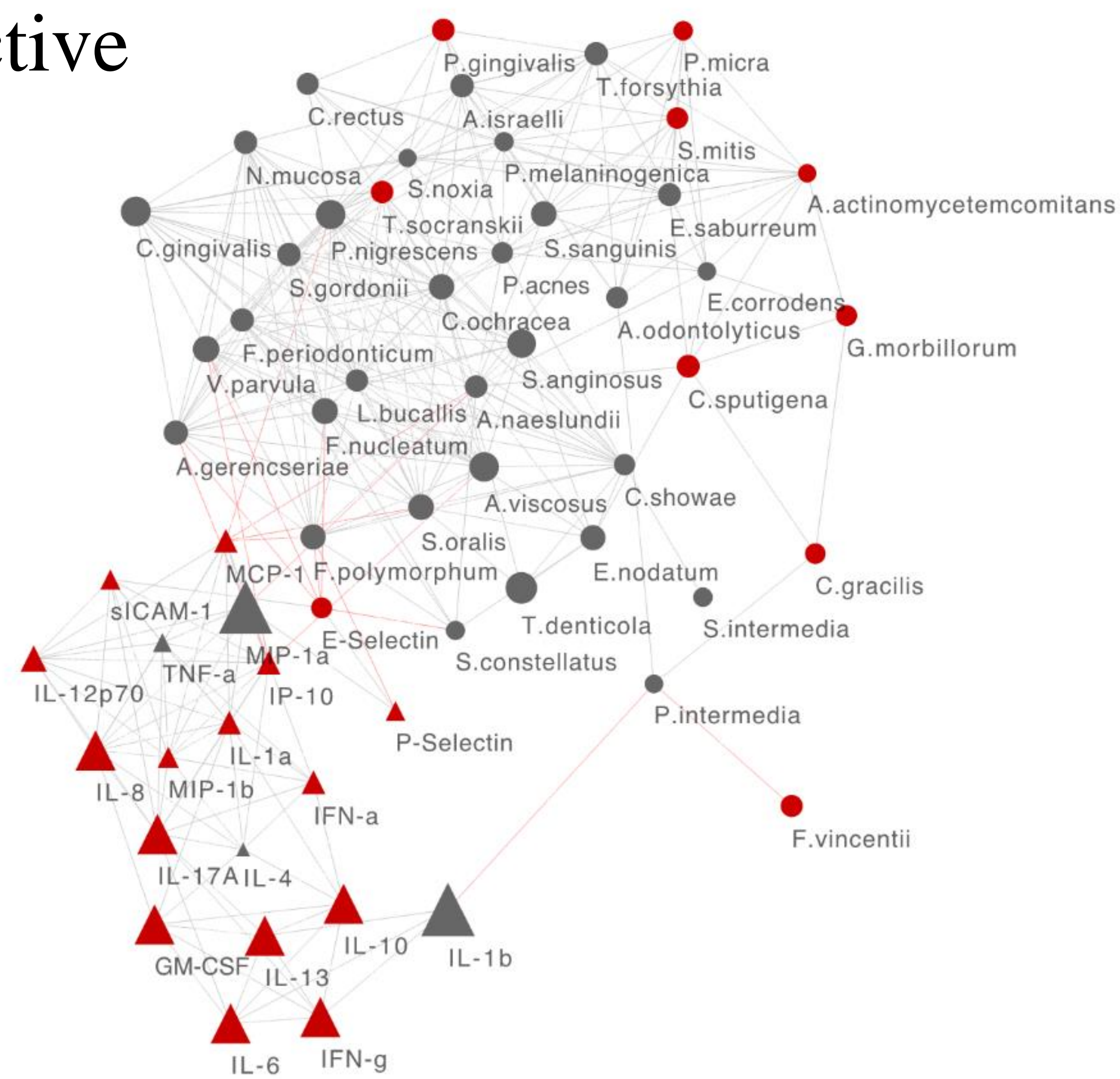

Supplement: Supplementary Figure 2 — Topological analysis of the co-occurrence networks among cytokines and bacterial species. The selected topological parameter illustrates the percentage connectivity of each bacterial species and cytokines within control, SLE-I, and SLE-A networks. [file Image_2.pdf]
