# Supplementary material for: Host-Microbial Interactions in Systemic Lupus Erythematosus and Periodontitis
Source: Front Immunol. 2019 Nov 12;10:2602. doi: 10.3389/fimmu.2019.02602 (PMC6861327; doi:10.3389/fimmu.2019.02602)
Supplement: Supplementary Table 1 — Comparison of cytokine levels between SLE and control groups. Significance was evaluated by the non-parametric Wilcoxon rank-sum test. Significant p-values (Benjamini & Hochberg adjusted) are highlighted in bold. [file Table_1.pdf]

Supplementary Table 1.

Cytokine	SLE-I vs Control	SLE-A vs Control	SLE-A vs SLE-I
IL-17A	**0.002298116**	0.79174097	0.2713651
IFN-a	**0.002298116**	0.65350477	0.2713651
sICAM-1	**0.002298116**	0.64712721	0.5034782
E-Selectin	0.241736248	**0.02290795**	0.5034782
IL-10	**0.014964202**	0.16080835	0.5034782
IFN-g	**0.003330498**	0.16080835	0.5034782
TNF-a	0.342512968	0.64712721	0.5034782
GM-CSF	**0.014964202**	0.23365079	0.5549938
MIP-1a	**0.014964202**	0.33591546	0.6287417
P-Selectin	0.911797181	0.31187418	0.6506653
IL-1b	**0.014964202**	0.44390222	0.6506653
IL-6	**0.004735736**	**0.03582795**	0.6506653
MCP-1	**0.017686529**	**0.03582795**	0.7017702
IL-12p70	**0.004735736**	0.13858759	0.7275608
IP-10	**0.020985207**	**0.02013466**	0.7765527
IL-13	0.176113576	0.44390222	0.8652679
IL-4	**0.002298116**	0.0563317	0.9074153
IL-8	**0.002298116**	**0.00938647**	0.9082021
MIP-1b	0.176113576	0.33591546	0.9228697
IL-1a	**0.038407413**	0.07118931	0.9228697
