# Supplementary material for: Host-Microbial Interactions in Systemic Lupus Erythematosus and Periodontitis
Source: Front Immunol. 2019 Nov 12;10:2602. doi: 10.3389/fimmu.2019.02602 (PMC6861327; doi:10.3389/fimmu.2019.02602)
Supplement: Supplementary Table 2 — Comparison of relative abundances of bacterial species between SLE and control groups with or without periodontitis. Significance was evaluated by the non-parametric Wilcoxon rank-sum test. Significant p-values (Benjamini & Hochberg adjusted) are highlighted in bold. [file Table_2.pdf]

Supplementary Table 2.

Bacterial species	Non-periodontitis			Periodontitis		
	SLE-I vs Control	SLE-A vs Control	SLE-A vs SLE-I	SLE-I vs Control	SLE-A vs Control	SLE-A vs SLE-I
C. ochracea	0.848339	**0.01492794**	**0.02985588**	0.74233937	**0.002085704**	**0.021098226**
S. intermedia	0.6890417	0.43581042	**0.04651062**	0.21095715	0.398088605	0.656457659
A. odontolyticus	0.6890417	0.24663775	**0.04651062**	0.90136976	0.636131812	0.501481566
F. polymorphum	0.848339	0.24663775	**0.04651062**	0.90136976	0.235219839	0.107299566
N. mucosa	0.6890417	0.10404839	0.05495359	0.33780588	0.526837352	0.122425841
T. socranskii	0.848339	0.21356998	0.05495359	0.14262184	0.971063279	0.189737104
E. saburreum	0.6890417	0.43581042	0.05495359	0.90136976	0.559984792	0.467374925
F. nucleatum	0.9121669	**0.04610472**	0.05495359	0.13744175	**0.008497623**	0.259268922
S. anginosus	0.890477	0.09317978	0.06091701	0.42258803	0.125491208	0.533258614
A. israelli	0.848339	0.24663775	0.06091701	0.82244714	0.080678122	0.118102598
V. parvula	0.848339	0.22927351	0.07298449	0.74233937	0.559984792	0.259268922
P. nigrescens	1	0.43743455	0.0814709	0.90136976	**0.008497623**	**0.040811022**
A. naeslundii	0.6890417	0.24663775	0.08645407	0.09403132	0.411939204	0.070552457
S. sanguinis	0.7778798	**0.01492794**	0.08645407	0.95123914	**0.049126921**	0.052398425
A. gerencseriae	0.7778798	**0.01686883**	0.08645407	**0.03353232**	0.225474714	**0.004816609**
P. micra	0.6890417	1	0.12448247	0.13744175	0.360561317	0.423826251
S. oralis	0.7778798	0.34461457	0.16632558	**0.01964188**	0.235219839	**0.011760555**
T. denticola	0.848339	**0.04610472**	0.16856461	0.69093326	0.243334593	0.118102598
C. gingivalis	0.9823701	0.24663775	0.16856461	0.13744175	**0.008497623**	0.322213951
E. nodatum	0.6890417	0.92541297	0.1786198	0.1663759	0.559984792	0.423826251
S. noxia	0.848339	0.24227481	0.1786198	**0.04182756**	0.676795945	0.063845606
C. showae	0.7778798	0.43743455	0.19546746	0.74233937	0.411939204	0.656812785
P. acnes	0.7778798	1	0.21232112	0.14262184	0.554029415	0.289936529
A. actinomycetemcomitans	0.9823701	0.43743455	0.27953117	0.75449744	0.987603002	0.446864473
A. viscosus	0.9793132	0.15779199	0.29902871	0.69133769	0.83228158	0.322213951
S. constellatus	0.7778798	1	0.39097177	0.14262184	0.559984792	0.322213951
P. melaninogenica	0.7778798	1	0.39097177	0.94997833	0.962745528	0.750274161
C. gracilis	0.7778798	0.6960154	0.40467312	0.19575829	0.962745528	0.070552457
T. forsythia	0.9823701	0.24663775	0.44836274	0.46158523	**0.008497623**	**0.040811022**
F. vincentii	0.6890417	0.43743455	0.44836274	0.13744175	0.158864459	0.583094407
C. rectus	0.848339	1	0.51577983	0.33780588	0.875148401	0.322213951
S. gordonii	0.9823701	0.40569571	0.51577983	0.14262184	**0.009475285**	0.515704124
S. mitis	0.9823701	0.44502385	0.5922647	0.67763882	0.254528182	0.871096819
F. periodonticum	0.6890417	0.15779199	0.61351131	0.13744175	0.093974649	0.572420173
C. sputigena	0.7778798	0.43581042	0.85049649	0.14262184	0.360561317	0.259268922
G. morbillorum	0.7778798	0.26749601	0.85703994	0.14262184	0.411939204	0.322213951
P. intermedia	0.8680691	0.43743455	0.87412417	0.94997833	0.656603895	0.750274161
P. gingivalis	0.848339	0.44648528	0.89520858	0.13744175	0.398088605	0.322213951
L. bucallis	0.848339	0.43743455	0.99546146	0.1663759	0.342512994	0.509484631
E. corrodens	0.848339	0.44648528	1	0.36337257	0.962745528	0.190158017
