# Supplementary material for: Host-Microbial Interactions in Systemic Lupus Erythematosus and Periodontitis
Source: Front Immunol. 2019 Nov 12;10:2602. doi: 10.3389/fimmu.2019.02602 (PMC6861327; doi:10.3389/fimmu.2019.02602)
Supplement: Supplementary Table 3 — Comparison of presence/absence of bacterial species between SLE and control groups with or without periodontitis. Significance was evaluated by the logistic regression model. Significant p-values (Benjamini & Hochberg adjusted) are highlighted in bold. [file Table_3.pdf]

Supplementary Table 3.

Bacterial species	Non-periodontitis			Periodontitis		
	SLE-I vs Control	SLE-A vs Control	SLE-A vs SLE-I	SLE-I vs Control	SLE-A vs Control	SLE-A vs SLE-I
C. ochracea	1	0.9146953	1	1	**0.002085704**	1
S. intermedia	1	1	0.8693035	0.4982064	0.398088605	0.7962139
A. odontolyticus	1	0.9146953	0.8693035	1	0.636131812	1
F. polymorphum	1	1	0.8693035	1	0.235219839	1
N. mucosa	1	0.8187469	1	1	0.526837352	1
T. socranskii	1	1	1	0.9813349	0.971063279	1
E. saburreum	1	1	1	1	0.559984792	1
F. nucleatum	1	1	1	1	**0.008497623**	0.7962139
S. anginosus	1	0.8187469	1	0.9813349	0.125491208	1
A. israelli	1	1	1	1	0.080678122	1
V. parvula	1	1	1	1	0.559984792	1
P. nigrescens	1	1	1	1	**0.008497623**	1
A. naeslundii	1	1	1	1	0.411939204	1
S. sanguinis	1	0.8187469	1	1	**0.049126921**	1
A. gerencseriae	1	1	1	1	0.225474714	1
P. micra	1	1	1	1	0.360561317	1
S. oralis	1	1	1	0.8203875	0.235219839	0.7252702
T. denticola	1	0.8187469	1	1	0.243334593	1
C. gingivalis	1	1	1	0.9813349	**0.008497623**	1
E. nodatum	1	1	1	0.4982064	0.559984792	1
S. noxia	1	0.8187469	1	1	0.676795945	1
C. showae	1	0.9146953	1	1	0.411939204	1
P. acnes	1	1	0.8693035	0.4031895	0.554029415	0.7252702
A. actinomycetemcomitans	1	1	1	1	0.987603002	1
A. viscosus	1	0.8187469	1	1	0.83228158	1
S. constellatus	1	1	1	1	0.559984792	1
P. melaninogenica	1	1	0.8948576	1	0.962745528	0.7252702
C. gracilis	1	1	1	0.4982064	0.962745528	0.7962139
T. forsythia	1	1	1	1	**0.008497623**	1
F. vincentii	1	0.8187469	1	1	0.158864459	0.7252702
C. rectus	1	1	0.8693035	1	0.875148401	1
S. gordonii	1	0.9146953	1	0.4982064	**0.009475285**	1
S. mitis	1	0.8187469	0.8693035	1	0.254528182	0.7909436
F. periodonticum	1	0.8187469	1	0.4982064	0.093974649	0.7962139
C. sputigena	1	0.8187469	0.8693035	0.9813349	0.360561317	0.8428107
G. morbillorum	1	0.8187469	1	1	0.411939204	1
P. intermedia	1	0.9146953	1	1	0.656603895	1
P. gingivalis	1	0.8187469	1	0.4982064	0.398088605	1
L. bucallis	1	0.9146953	1	0.4982064	0.342512994	0.7962139
E. corrodens	1	0.8187469	1	0.4982064	0.962745528	1
