# Supplementary material for: Host-Microbial Interactions in Systemic Lupus Erythematosus and Periodontitis
Source: Front Immunol. 2019 Nov 12;10:2602. doi: 10.3389/fimmu.2019.02602 (PMC6861327; doi:10.3389/fimmu.2019.02602)
Supplement: Supplementary Table 4 — Comprehensive demographics table and clinical comparisons of SLE and control subjects with chronic or non-chronic periodontitis. [file Table_4.pdf]

Supplementary Table 4. Correlation of Systemic lupus erythematosus (SLE) with Chronic/Non-chronic Periodontitis (CP/Non-CP).

		Control				SLE-Inactive							SLE-Active							p-value *
		Total	Non-CP	CP	Non-CP x CP within group ϕ	Total	Non-CP	CP	Non-CP x CP within group ϕ	**Total**: SLE-Inactive & **Control** group ϕ	**Non-CP**: SLE-Inactive & **Control** group ϕ	**CP**: SLE-Inactive & **Control** group ϕ	Total	Non-CP	CP	Non-CP x CP within group ϕ	**Total**: SLE-Active & **Control** group ϕ	**Non-CP**: SLE-Active & **Control** group ϕ	**CP**- SLE: Active & **Control** group ϕ	Control x SLE-Inactive x SLE-Active group
**DEMOGRAPHICS**																				
**No. of Subjects** [c]		31	8 (25.80%)	23 (74.19%)		29	9 (31.03%)	20 (68.97%)					31	13 (41.94%)	18 (58.06%)					
**Gender**											100% Female Subjects									
**Age** (years)		37.42 ± 8.72	34.25 ± 9.04	38.52 ± 8.53	0.239	37.69 ± 8.97	35.89 ± 9.16	38.50 ± 9.00	0.4783	0.9062	0.7161	0.9936	32.58 ± 8.91	31.38 ± 9.92	33.44 ± 8.30	0.5348	0.0348*	0.5147	0.0631	0.0448 *
**Ethinicity** [c]	Black	8 (25.80%)	2 (0.06%)	6 (0.19%)		3 (10.34%)	1 (3.45%)	2 (6.90%)					7 (22.58%)	0	7 (22.58%)					
	Mixed	16 (51.61%)	4 (12.90%)	12 (38.71%)		18 (62.07%)	7 (24.14%)	11 (37.93%)					20 (64.52%)	9 (29.03%)	11 (35.48%)					0.3929 [b]
	White	7 (22.58%)	2 (6.45%)	5 (16.13%)		8 (27.59%)	1 (3.45%)	7 (24.14%)					4 (12.90%)	4 (12.90%)	0					
**ORAL HEALTH- PERIODONTAL PARAMETERS**																				
**Teeth #**		23.32 ± 4.56	25.00 ± 3.16	22.74 ± 4.87	0.2328	23.72 ± 5.40	26.56 ± 1.67	22.45 ± 6.03	0.0568	0.7562	0.2162	0.8629	24.06 ± 4.13	25.62 ± 2.79	22.88 ± 4.77	0.0769	0.5044	0.6455	0.8919	0.8251
**Bleeding Points**		23.77 ± 20.36	11.88 ± 9.14	27.91 ± 21.66	0.0533	14.66 ± 12.32	13.89 ± 15.97	15.00 ± 10.77	0.8269	0.0418*	0.7584	0.0202*	15.84 ± 21.61	12.15 ± 17.41	18.50 ± 24.34	0.4291	0.1419	0.9672	0.1985	0.1225
**BOP (%)**		17.33 ± 14.90	8.09 ± 6.13	20.55 ± 15.78	0.0394*	11.38 ± 11.92	8.82 ± 10.66	12.54 ± 12.53	0.4476	0.0944	0.8664	0.0754.	11.19 ± 14.62	7.98 ± 10.80	13.52 ± 16.78	0.3059	0.1069	0.9793	0.1763	0.1513
**Stainned sites**		55.58 ± 21.68	46.25 ± 13.33	58.83 ± 23.27	0.1611	55.34 ± 20.45	41.33 ± 16.58	61.65 ± 19.13	0.0105*	0.9656	0.5143	0.669	52.58 ± 20.06	45.85 ± 17.16	57.44 ± 21.03	0.1134	0.5738	0.9554	0.8451	0.8201
**Plaque Index**		60.05 ± 20.90	46.6 ± 14.37	64.70 ± 21.02	0.0325*	61.69 ± 24.91	39.13 ± 16.60	71.84 ± 21.20	0.0004***	0.7831	0.3387	0.2764	56.65 ± 23.62	45.79 ± 20.14	64.48 ± 23.30	0.0270*	0.5503	0.9201	0.9733	0.6900
**PD (mean)**		2.35 ± 0.35	2.17 ± 0.19	2.42 ± 0.37	0.0813	2.41 ± 0.27	2.31 ± 0.31	2.45 ± 0.25	0.2128	0.5165	0.2737	0.7517	2.39 ± 0.40	2.37 ± 0.53	2.40 ± 0.29	0.8471	0.7405	0.323	0.8502	0.8375
**PD ≥ 4 (n)**		10.52 ± 12.94	7.50 ± 6.19	11.57 ± 14.55	0.4534	9.79 ± 7.13	4.00 ± 2.35	12.40 ± 7.04	0.0018**	0.7616	0.1351	0.8164	10.16 ± 8.65	6.54 ± 3.50	12.78 ± 10.30	0.0456*	0.8994	0.6523	0.7663	0.9611
**CAL (mean)**		0.61 ± 0.33	0.40 ± 0.16	0.68 ± 0.35	0.0442*	1.00 ± 1.16	0.80 ± 1.14	1.09 ± 1.19	0.5388	0.0731	0.3453	0.1155	0.72 ± 0.53	0.41 ± 0.20	0.94 ± 0.59	0.0051**	0.3316	0.8965	0.0905	0.1157
**CAL ≥ 4 (n)**		5.65 ± 4.48	3.13 ± 2.80	6.52 ± 4.66	0.0633	8.31 ± 9.44	4.00 ± 6.86	10.25 ± 9.94	0.0997	0.1632	0.7415	0.1152	7.55 ± 9.11	2.31 ± 2.02	11.33 ± 10.36	0.0044**	0.3005	0.4457	0.0536	0.4104
**Classification of PD**	Non-periodontites Control	8 (25.81%)	8	0		9 (31.03%)	9	0					13 (41.94%)	13	0					
	Mild periodontitis	11 (35.48%)	0	11		8 (27.58%)	0	8					9 (29.03%)	0	9					
	Moderate periodontitis	9 (29.03%)	0	9		6 (20.69%)	0	6					4 (12.90%)	0	4					0.5761[b]
	Severe periodontitis	3 (9.67%)	0	3		6 (20.69%)	0	6					5 (16.13%)	0	5					
**SYSTEMIC HEALTH- SLE PARAMETERS**																				
**Time with SLE** (years)		0	0	0	N/A	11.90 ± 6.91	9.11 ± 5.35	13.15 ± 7.28	0.1487	<0.0001 ****	0.0002***	<0.0001 ****	6.68 ± 4.06	6.31 ± 4.57	6.94 ± 3.77	0.6741	<0.0001 ****	0.0010**	<0.0001 ****	<0.0001 ****
**SLEDAI**		0	0	0		1.07 ± 0.99	1.11 ± 1.05	1.05 ± 0.99	0.8819	<0.0001 ****	0.0095**	<0.0001 ****	7.29 ± 4.31	8 ± 5.70	6.78 ± 3.02	0.4449	<0.0001 ****	0.0009***	<0.0001 ****	<0.0001****
**SLEDAI subgroups** [c]	SLEDAI (0)	31 (100%)	8	23		13 (44.83%)	4	9					0	0	0					
	SLEDAI (1-5)	0	0	0		16 (55.17%)	5	11					14 (45.16%)	7	7					
	SLEDAI (6-8)	0	0	0		0	0	0					9 (29.03%)	2	7					
	SLEDAI (9-19)	0	0	0		0	0	0					7 (22.58%)	3	4					
	SLEDAI (> 20)	0	0	0		0	0	0					1 (3.23%)	1	0					

[a] Values represent mean ± SD or percentage (%); Statistical difference (ϕ p-values) within group comparision were calculated by Two tailed-Unpaired t-test assuming both population having same SD and (* p-values) among group comparisions were calculated by one-way ANOVA for numerical features; [b] Chi-square test for categorical features; [c] n (% within group).

Abbreviations : SLE-Systemic lupus erythematosus; CP-Chronic periodontitis; BOP-Bleeding on probing; PD-Probing depth; CAL-Amount of clinical attachment loss; SLEDAI-Systemic lupus erythematosus disease activity index.
